# Prescribed drugs and comorbidities as risk factors for Torsades de Pointes arrhythmia: a Swedish population-based cohort study

**DOI:** 10.1007/s00228-025-03990-9

**Published:** 2026-01-19

**Authors:** Marine L. Andersson, Johan Fastbom, Bengt Danielsson, Eva Wikström, Marja-Liisa Dahl, Karolina Nowinski

**Affiliations:** 1 Department of Laboratory Medicine and Department of Clinical Pharmacology, Karolinska Institutet and Karolinska University Hospital, Stockholm, Sweden; 2 Aging Research Center, Karolinska Institutet and Stockholm University, Stockholm, Sweden

**Keywords:** Torsade de pointes arrhythmia, QT prolongation, Risk factors, Sex differences, Comorbidities, Clinical decision support systems (CDSS)

## Abstract

**Purpose:**

Drug-induced long QT interval syndrome (LQTS) is a risk for Torsade de Pointes (TdP) arrhythmia. The purpose was to investigate patients diagnosed with TdP in relation to dispensed risk drugs, the presence of comorbidities, and sex-differences.

**Methods:**

All patients with a first episode of a registered TdP diagnosis in the National Patient Register between 2006 and 2018 were included in this register-based cohort study. Comorbidities within five years and medications dispensed within 90 days prior to TdP diagnosis were retrieved. Drugs were classified for their risk to cause TdP (“risk drugs”) using two different Clinical Decision Support Systems (CredibleMeds and Janusmed).

**Results:**

The cohort consisted of 762 patients (50% females) with TdP, median age 72 years. A majority (59%) were dispensed at least one risk drug and 338 patients (44%) had at least one risk diagnosis for TdP. Concomitant use of several risk drugs was common. Prior to TdP, 558 patients (73%) had at least one risk diagnosis and/or were dispensed at least one risk drug. More females than males used antidepressants (26% vs 12%, *p* < 0.01) although the total use of risk drugs were similar in females compared to males. More males than females had risk diagnoses for TdP (53% vs. 36%, *p* < 0.004).

**Conclusion:**

A majority of patients with TdP had been dispensed risk drugs and/or had risk diagnoses. Sex-differences in dispensed drugs and risk diagnoses were identified. Prescription drugs, underlying risk diagnoses and sex should be incorporated into the risk assessment for TdP.

**Graphical abstract:**

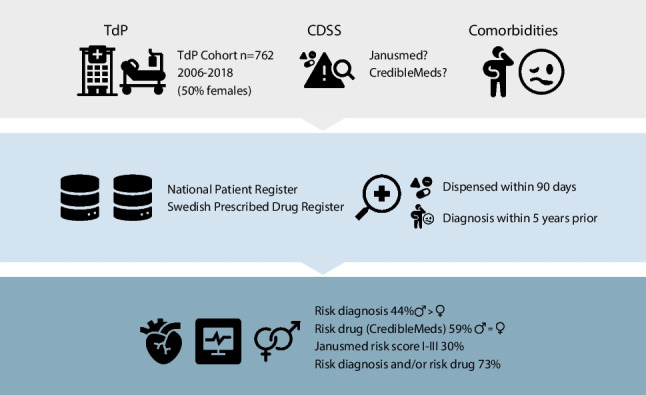

**Supplementary Information:**

The online version contains supplementary material available at 10.1007/s00228-025-03990-9.

## Introduction

Drug-induced long QT interval syndrome (LQTS) can cause life-threatening Torsades de Pointes (TdP) ventricular arrhythmia. Known risk factors include age > 65 years, bradycardia, acute myocardial infarction, electrolyte abnormalities, heart failure, history of drug-induced TdP and female sex. Furthermore, conditions leading to elevated plasma concentrations of QT prolonging drugs – such as drug-drug interactions, and inadequate dose adjustment of renally eliminated or hepatically metabolized drugs in patients with kidney or liver disease, respectively – can increase the risk. Certain individuals have increased susceptibility to drug-induced LQTS and TdP due to genetic predisposition, such as mutations in hERG channel genes, or altered expression of other genes involved in the regulation of cardiac repolarization [1, 2].

Clinical decision support systems (CDSS) are designed to help prescribers assess the risk for TdP associated with prescription drugs. CredibleMeds is an online resource identifying drugs associated with QT prolongation and TdP [3]. The Swedish Janusmed Risk Profile (Janusmed) is a CDSS accessible within electronic healthcare records and online. It estimates the risk of QT prolongation/TdP of individual prescription drugs and provides an algorithm-based risk score when combining several drugs [4].

In a previously published national register-based cohort study in Sweden, 762 patients with a diagnosis of TdP were identified of whom 410 used drugs classified by CredibleMeds as being associated with an increased risk of TdP. Antidepressants and antiarrhythmics were the most frequently prescribed classes of “risk drugs” associated with TdP [5]. Another Swedish national study showed that mortality rates among elderly were higher among patients prescribed antidepressants classified as known or possible risk drugs compared to antidepressants classified as non-risk drugs (using CredibleMeds) [6].

The objective of the present register-based study was to further investigate the patients diagnosed with TdP in relation to: 1) dispensed risk drugs, as assessed by two different clinical decision support systems (CredibleMeds and Janusmed), 2) the presence of comorbidities and risk diagnoses, and 3) sex-differences regarding comorbidities and use of risk drugs. Our long-term goal is to raise awareness about the importance of personalized and tailored approaches in the risk assessment of TdP.

## Methods

### Study population

All persons in Sweden with a diagnosis of TdP (ICD-10 code I472.C) between January 1, 2006 and December 31, 2017 were included in the study cohort [6]. For patients with more than one TdP diagnosis registered, only the first occasion was included. The data underlying this article are available in the article and in its online supplementary material.

### Registries

The Swedish National Board of Health and Welfare is the holder of all registers used in this study. Data were collected from the National Patient Register (NPR), the Swedish Prescribed Drug Register (SPDR) and the National Cause of Death Register (NCDR). The registries were linked at an individual level by the Swedish National Board of Health and Welfare.

The NPR covers all inpatient care in Sweden since 1987 and specialized outpatient care since 2001. Primary care data is not included in the NPR. Primary and secondary diagnoses are coded according to the International Classification of Diseases (ICD-10) [7]. The hospital admission and discharge dates are registered in the NPR, but the exact date of the occurrence of the diagnoses during the hospital stay is not specified. Report of the sex of individuals (female or male) in the NPR is extracted from medical files and is defined as the legal gender.

The SPDR contains patient level data on all prescribed drugs dispensed at Swedish pharmacies since July 2005, including information on trade name, substance name, Anatomical Therapeutic Chemical (ATC) classification code, drug form, strength, dispensed amount, dosage, and prescriber details [8]. NCDR includes all deaths in Sweden registered since 1952. Causes of death and contributing causes of death are coded according to ICD-10 [9].

### Clinical decision support systems (CDSSs)

All prescription medications (drugs) dispensed within 90 days prior to the hospital care episode for TdP in a previous study were included in the analysis. For each individual patient, drugs were assessed for their risk of QT interval prolongation and TdP using two different CDSSs, CredibleMeds (https://www.crediblemeds.org/, 31 October 2018) and Janusmed Risk Profile (https://www.janusmed.se/, access date November 2019). These two CDSS systems have different principles for risk classification.

#### CredibleMeds

CredibleMeds (Arizona Center for Education and Research on Therapeutics, AZCERT) is a well-established source that provides information on the risk of QT prolongation or TdP for individual drugs. Each drug included is classified as “known risk”, “possible risk” (if the drugs is taken as recommended) or “conditional risk” [3]. Drugs classified as conditional risk are drugs that under certain circumstances (e.g. interactions, electrolyte disturbances) may contribute to the development of TdP.

#### Janusmed risk profile 

Janusmed Risk Profile (Janusmed) is a CDSS available online for all prescribers in Sweden since autumn 2022 (in Stockholm since 2017) and is embedded in the electronic health record system in some regions in Sweden [10]. It provides information on nine different risk properties for all drugs (at substance level) and can be used to assess additive effects and the risk for pharmacodynamic drug interactions. One of the nine properties is risk for QT prolongation. All drug substances in the database have been graded on a scale of 0–3 with respect to the risk for QT prolongation and TdP, with 3 indicating the highest risk, 1 indicating the lowest risk and 0 indicating no known risk [10]. As an example, drugs with the highest risk grade include antiarrhythmics and methadone. At the time of the present analysis (November 29, 2019), approximately 1600 pharmaceutical substances had been classified in the Janusmed database regarding the risk of QT prolongation, with 148 as grade 1, 59 as grade 2, 13 as grade 3 and the remaining 1380 substances as grade 0.

Janusmed includes an algorithm which takes the classifications of all prescribed drugs for the particular individual patient into account. A sum of the total risk for drug-induced QT prolongation is calculated and generates a Janusmed risk score. A risk score of I, II or III indicates an increased risk for a combination of drugs. The assumption behind the algorithm is that simultaneous use of multiple drugs with QT-prolonging properties increases the risk for QT prolongation and TdP to a larger extent than use of only one QT-prolonging drug [11]. If the sum of the grades of the drugs is 2 or 3 the risk is classified as “slightly increased” (risk score I). A sum of 4 indicates a “moderately increased risk” (risk score II) and a sum of 5 or higher a “highly increased” risk (risk score III) for QT prolongation. The use of at least two drugs classified as grade 1 or one drug classified as grade 2 is a minimum criterion for classifying a patient as having an increased risk. Additionally, the presence of at least one drug classified as grade 3 (e.g. sotalol) automatically results in a classification of “highly increased” risk even without other contributing medications.

#### Comparison between Janusmed and CredibleMeds

The two distinct CDSSs Janusmed and CredibleMeds are not directly comparable. In CredibleMeds a drug is classified as associated with known risk if there is enough documentation to support that it can cause TdP, even if the risk would be rather low (i.e. erythromycin). In Janusmed the risk of each substance is graded according to risk for TdP as perceived in the literature, and hence drugs such as erythromycin have a lower grade (grade 2) compared to for example antiarrhythmic drugs (grade 3). The sum of the grades of each drug is translated into a summed risk score (I-III) for that individual taking all concomitant drugs into account. Also, Janusmed has no category similar to the “conditional risk” in CredibleMeds.

### Dispensed medication and classification of risk

A “risk-drug” was defined as a drug associated with QT prolongation and/or TdP according to CredibleMeds and/or Janusmed. Specifically, a” risk drug” was classified as having a “known risk”, “possible risk” or “conditional risk” according to CredibleMeds and/or a drug with a risk grade of 1, 2 or 3 according to Janusmed. Janusmed generated a risk score for each individual by linking data on dispensed drugs with Janusmed data and its algorithm for QT prolongation (described in section Clinical decision support systems). Topical drugs were not included since they were not included in the Janusmed at the time of the analysis.

To obtain a crude estimate of the odds of using a riskdrug in our TdP cohort, we used data on dispensed medication in the whole Swedish population aged 65 years and between 2006 and 2017 retrieved from the online database of the Swedish National Board of Health and Welfare. Odds ratios (OR) were calculated as the odds of using a drug in the TdP cohort divided by the odds of it being used (median number of patients per 1000 inhabitants) in the Swedish population aged 65 or older.

### Comorbidities

The presence of comorbidities both during the hospital care episode and in the five years preceding it were analysed. Comorbidities relevant for identifying heart disease and other risk factors according to CredibleMeds and the literature were converted into ICD codes and retrieved from the NPR [1, 3]. Comorbidities were retrieved: a) within 5 years prior to the admission date for the hospital care episode during which TdP was diagnosed and b) during the hospital care episode when TdP was diagnosed. A full list of retrieved diagnoses is provided in the supplementary material.

The following risk diagnoses during the five years prior to the hospital care episode were included in the analysis: heart failure, cardiomyopathies, acute and chronic coronary syndromes, renal failure, liver disease, LQTS, the presence of ventricular arrhythmia and cardiac arrest [1]. The following conditions during the hospital care episode of TdP were included: heart failure, cardiomyopathies, renal failure, cerebrovascular diseases, acute coronary syndrome, ventricular arrhythmias, sepsis, poisoning, atrioventricular (AV) block II and III, and LQTS. To describe comorbidities related to structural heart disease the following comorbidities were included: heart failure, cardiomyopathies, angina pectoris, acute and chronic coronary syndromes, and ventricular arrhythmia.

### Statistical analysis

The number of patients with an increased risk according to Janusmed Risk Profile was compared between males and females using Fisher’s exact test. *P* < 0.05 was set as limit for statistical significance. The odds ratio (OR) and 95% confidence interval (95% CI) for being dispensed a specific drug were calculated using Fisher’s exact test. Statistical analysis was performed using R version 4.3.1.

## Results

### Patient characteristics

762 patients (50% females) with a diagnosis of TdP between 2006 and 2017 were identified. Median age was 72 years, and the majority (72%) was 65 years or older. The five most common diagnoses registered during the five-year period before the occurrence of TdP were hypertension (36%), atrial fibrillation/atrial flutter (28%), acute and chronic coronary syndromes (24%), and heart failure (22%) (Table 1). Diagnoses registered at the hospital care episode when TdP occurred are shown in, supplementary material Table 1. Mortality within one week after diagnosis of TdP was 4% (*n* = 31), within 30 days 8% (*n* = 61), and within 90 days 11% (*n* = 87).

**Table 1:** Characteristics of the 762 patients (383 females and 379 males) with Torsades de Pointes (TdP) arrhythmia

N (%)	Females *n* = 383	Males *n* = 379	All *n* = 762
Age mean, median (IQR, range) (years)	74, 70 (64–83, 4–100)	70, 69 (63–78, 19–95)	72, 69 (63–81, 4–100)
Age 0–64 years	103 (27)	110 (29)	213 (28)
Age 65–79 years	144 (38)	184 (49)	328 (43)
Age ≥ 80 years	136 (36)	85 (22)	221 (29)
Number of drugs dispensed, median (IQR)	6 (3–10)	6 (2–9)	6 (2–9)
Atrial fibrillation or flutter	91 (24)	122 (32)	213 (28)
Hypertension	135 (35)	142 (37)	277 (36)
Heart failure	71 (19)	99 (26)	170 (22)
Cardiomyopathies	17 (4)	17 (4)	34 (4)
Angina pectoris	28 (7)	28 (7)	56 (7)
Acute coronary syndrome	42 (11)	72 (19)	114 (15)
Chronic coronary syndrome	52 (14)	85 (22)	137 (18)
Cerebrovascular diseases	23 (6)	40 (11)	63 (8)
Renal failure	19 (5)	35 (9)	54 (7)
Chronic kidney disease	9 (2)	10 (3)	19 (2)
Chronic obstructive pulmonary disease	24 (6)	19 (5)	43 (6)
Liver disease	4 (1)	6 (2)	10 (1)
Rheumatic diseases	11 (3)	17 (4)	28 (4)
Vascular disease	17 (4)	25 (7)	42 (6)
Cardiac arrest	14 (4)	25 (7)	39 (5)
Ventricular fibrillation	14 (4)	19 (5)	33 (4)
Long QT Syndrome (LQTS)	12 (3)	3 (1)	15 (2)
Supraventricular arrhythmia	18 (5)	15 (4)	33 (4)
Premature depolarization	5 (1)	4 (1)	9 (1)
Paroxysmal tachycardia	10 (3)	4 (1)	14 (2)
Reentry ventricular arrhythmia and/or paroxysmal ventricular tachycardia	30 (8)	45 (12)	75 (10)
AV block II and III	16 (3)	12 (3)	28 (3)
Fascicular block, bundle-branch block, intraventricular block, pre-excitation	8 (2)	8 (2)	16 (2)
Sick sinus syndrome	8 (2)	6 (2)	14 (2)

Comorbidities were defined as a presence of an ICD-10 code during the 5 years preceding the occurrence of TdP diagnosis. Several diagnoses could coexist in the same patient. Unless stated otherwise, the numbers refer to n (%). *IQR* interquartile range

### Dispensed drugs in patients with TdP

Patients were dispensed a median of six drugs (interquartile range, IQR, 2–9 drugs) within 90 days prior to the date of TdP diagnosis. Acetylsalicylic acid, furosemide and metoprolol were the top three most common drugs (Table 3, supplementary material). Seventy percent of patients with TdP were using one or more cardiovascular drugs. Only 6.6% of patients where dispensed a cancer drug. Eigthy-six patients (11%) were not dispensed any drug within 90 days prior to TdP.

Antidepressants and antiarrhythmics were among the most common risk drugs dispensed. Antiarrhythmic drugs (class I and III antiarrhythmics) were used in 77 patients (10%). The most common risk drugs are listed in Table 2. A higher proportion of patients with TdP compared to the Swedish population 65 years or older had been dispensed sotalol (OR 7.3), amiodarone (OR 7.5), olanzapine (OR 6.6) or citalopram (OR 1.7). There was no significant difference in the proportion of patients with mirtazapine, hydroxyzine, sertraline, loperamide or alfuzosin between the TdP-cohort and the Swedish population. Ciprofloxacin was dispensed less frequently in patients with TdP (OR 0.48).

**Table 2:** Patients with Torsade de Pointes arrhythmia (TdP) and the 12 most common dispensed drugs associated with risk for QT prolongation and TdP

Substance	N	Janusmed classification	Credible Meds classification	Odds ratio (95% C.I)	*p* value
citalopram	80	2	known	1.71 (1.23–2.41)	*p* < 0.01
sotalol	38	3	known	7.30 (3.27–19.2)	*p* < 0.001
mirtazapine	32	1	possible	1.05 (0.66–1.68)	*p* = 0.91
hydroxizine	23	2	conditional	1.07 (0.61–1.88)	*p* = 0.89
amiodarone	22	3	known	7.47 (2.23–39.1)	*p* < 0.001
sertraline	19	1	conditional	1.00 (0.55–1.83)	*p* = 1
ciprofloxacin	17	2	conditional	0.48 (0.27–0.82)	*p* < 0.01
alfuzosin	14	1	possible	1.34 (0.69–2.66)	*p* = 0.44
loperamide	14	0	conditional	1.06 (0.51–2.20)	*p* = 1
olanzapine	10	1	possible	6.57 (1.48–60.12)	*p* < 0.01
metoclopramide	10	1	conditional	2.20 (0.72–7.41)	*p* = 0.13
buprenorphine	10	0	possible	3.31 (0.95–14.0)	*p* = 0.054

Risk classification of drugs was performed using Janusmed and CredibleMeds. Odds ratios signifies the odds of using the drug in individuals with TdP divided by the odds of the drug used in the Swedish population aged 65 or older. Drugs dispensed to ten or more patients were included in the table

### Dispensed risk drugs in patients with TdP according to two different CDSSs

**Fig. 1:**
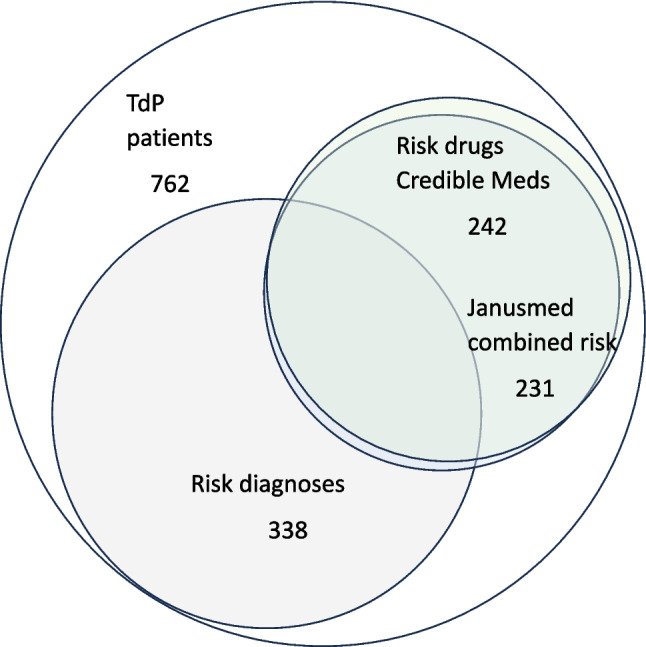
The circles represent the number of patients with Torsade de Pointes arrhythmia (TdP), risk diagnoses and risk drugs. Risk drugs were defined according to classification with Credible Meds (known or possible risk) and Janusmed Risk Profile (risk associated with combined drug treatment)

A majority of patients (*n* = 450, 59%) was dispensed at least one risk drug according to the CredibleMeds classification, including drugs with conditional risk. Almost a third of patients (*n* = 242; 32%) were dispensed any risk drug classified as known or possible risk (Table 3, Fig. 1). According to Janusmed classification (which does not include the conditional risk category) 288 patients (38%) were dispensed at least one risk drug.

**Table 3:** Number of patients (n) with Torsade de Pointes arrhythmia (TdP) with and without risk diagnoses that were dispensed risk drugs

Classification of dispensed drugs	Risk diagnoses (*n* = 338) n (%)	No risk diagnoses (*n* = 424) n (%)	All patients (*n* = 762) n (%)
Credible Meds classification of individual drugs			
Any risk drug (known, possible and/or conditional risk)	231 (68)	219 (52)	450 (59%)
Any risk drug (known and/or possible risk)	111 (33)	131 (31)	242 (32%)
≥ 1 drug known risk	92 (27)	100 (24)	
≥ 1 drug possible risk	35 (10)	55 (13)	
≥ 1 drug conditional risk	202 (60)	164 (39)	
Janusmed classification of individual drugs			
Any risk drug 1–3	135 (40)	153 (36)	288 (38%)
Janusmed algorithm of combined risk for TdP			
Janusmed combined risk	111 (33%)	120 (28%)	231 (30%)
High risk	53	43	
Moderate risk	4	11	
Slight risk	54	66	

The percentage (%) refers to the proportion of patients who were dispensed risk drugs (classified according to Credible Meds or Janusmed) relative to those with risk diagnoses, without risk diagnoses or relative to all patients. A risk diagnosis was defined as the presence of heart failure, cardiomyopathies, acute and chronic coronary syndrome, renal failure, liver disease, Long QT syndrome (LQTS), ventricular arrhythmia or cardiac arrest up to five years before the hospital care episode of TdP

Coadministration of several risk drugs was relatively common. Among patients using a drug with known risk, almost one third (30%) were also dispensed another drug classified as known or possible risk, while an additional 43% were using a drug with conditional risk (using CredibleMeds). Almost one third of patients (*n* = 231, 30%) were prescribed a combination of drugs that resulted in Janusmed risk score I-III (13% had a high risk, 2% had a moderately increased risk and 16% had a slightly increased risk) (Table 3).

### Comorbidities associated with increased risk for TdP (“risk diagnoses”)

Almost half of the patients (*n* = 338) had one or more risk diagnoses (heart failure, cardiomyopathies, acute and chronic coronary syndrome, renal failure, liver disease, LQTS, the presence of ventricular arrhythmia or cardiac arrest) registered during the five years before the hospital care episode of TdP. Twenty-three percent of patients (*n* = 174) had at least two risk diagnoses.

Cardiovascular diseases related to structural heart disease (heart failure, cardiomyopathies, ischemic heart diseases, ventricular fibrillation or tachycardia) were present in 314 patients (41%). Prior ventricular arrythmia and/or cardiac arrest were present in 99 patients (13%) (ventricular tachycardia *n* = 75, and/or ventricular fibrillation *n* = 33 and/or cardiac arrest *n* = 39). The diagnosis of LQTS was present in fifteen patients (Table 1).

### Prevalence of risk diagnoses and use of risk drugs prior to TdP

Approximately one third of patients with risk diagnoses also used at least one risk drug (classified as known or possible risk using CredibleMeds or Janusmed risk score I-III). Another third of patients with risk diagnoses used a drug with conditional risk (using CredibleMeds). Patients with risk diagnoses seemed to use risk drugs to a larger extent compared to patients without risk diagnoses. Even in those without risk diagnoses approximately 50% of patients used risk drugs including drugs with conditional risk (Table 3).

A majority of patients (*n* = 558, 73%) with a registered TdP diagnosis had - prior to the occurrence of TdP - been dispensed at least one risk drug with risk for QT prolongation (known, possible or conditional risk using Credible Meds) (“risk drug”) and/or had comorbidities associated with risk for TdP (“risk diagnosis”). Twenty-seven percent had neither known risk diagnoses nor risk drugs. Using Janusmed Risk Profile, 458 patients (60%) had at least one risk diagnosis and/or any risk drug (risk category 1, 2 or 3) prior to TdP (Fig. 1).

### Sex-differences in risk diagnoses and use of risk drugs prior to TdP

Females were dispensed antidepressants to a larger extent than males (26% vs 12%, *p* < 0.01) (Supplementary material, Table 3). Although numerically more males than females used cardiovascular drugs (73% versus 68%), the difference was not statistically significant. There was no statistically significant difference between females and males in total dispensed risk drugs. Specifically, 165 (43%) females and 174 (46%) males were dispensed medication classified as known, possible or conditional risk (CredibleMeds) and/or increased risk score (Janusmed). Additionally, there was no statistically significant difference between females and males in dispensed drugs classified as “known risk for TdP” according to Credible Meds (28% vs 22%, *p* = 0.095) or as increased risk score (Janusmed) (33% vs 28%, *p* = 0.20).

Risk diagnoses (heart failure, cardiomyopathies, acute and chronic coronary syndromes, renal failure, liver disease, LQTS, a history of ventricular arrhythmia or cardiac arrest) were more common in males compared to females (53% vs. 36%, *p* < 0.004). Heart disease (heart failure, cardiomyopathies, angina pectoris, acute and chronic coronary syndromes, ventricular arrhythmia) was also more prevalent in males compared to females (48% vs 34%, *p* < 0.001). However, long QT syndrome was more frequently observed in females (12 vs 3 individuals, *p* = 0.033).

## Discussion

This is one of the largest studies of patients with TdP arrhythmia, including 762 individuals in a population of 9–10 million over a 12-year period. The main finding of our study was that 73% of patients had been dispensed at least one drug and/or had at least one diagnosis associated with increased risk for arrhythmia before the occurrence of TdP. Use of several risk drugs was common. Furthermore, sex differences in dispensed drugs and risk diagnoses were identified. More males than females had risk diagnoses for TdP except for LQTS which occurred more frequently in females. A higher proportion of females than males used antidepressants, however no significant difference was observed between females and males in the use of any risk drug. The results are in accordance with a study observing several risk factors among 249 patients with drug-related TdP from case reports showed that 80–90% of patients had one or several risk factors for TdP such as potential drug interactions (defined as administration of two or more drugs that prolong the QT interval or compete for the same metabolic pathway) and heart disease (myocardial infarction, heart failure, valvulopathy, or cardiomyopathy) [12].

The incidence of TdP in our study would translate to approximately 6 cases of TdP per million individuals annually. However, the true incidence of TdP is probably much higher since our data come from diagnoses registered in hospital care. The prevalence of TdP outside hospital care is not fully known. In comparison, a previous study reported of an age-standardized incidence of symptomatic drug-induced LQTS among 51 hospitals in Berlin (2.9 million inhabitants) to be 2.5 per million per year for males and 4.0 per million per year for females during a 3-year period [1, 13].

A high degree of agreement was found between the two distinct CDSS systems used in this study regarding classifications for known or possible risk in CredibleMeds and Janusmed; however, Janusmed does not include drugs with conditional risk to the same extent. A key difference between the two CDSSs is that Janusmed is integrated into electronic health care records providing an overview of the individual patient’s complete medication list including both pharmacokinetic and pharmacodynamic drug-drug interactions. Citalopram, sotalol and amiodarone were among the top five most dispensed risk drugs in our study cohort. The use of citalopram, escitalopram and amiodarone was higher among patients with TdP compared to the Swedish population aged 65 years and older. Although the cohorts were not matched, the analysis provided a crude comparison of drug use between the study cohort and the general population. These results are consistent with a previous retrospective study of over 400,000 geriatric inpatients which found that citalopram, escitalopram, and amiodarone were the most frequently prescribed drugs associated with risk for QT prolongation [14].

Prescription of multiple risk drugs was relatively common in our study. Almost 75% of patients who were dispensed a drug with known risk, were concomitantly dispensed another risk drug (classified as known, possible, or conditional risk). The observation that many patients used multiple QT-prolonging drugs concomitantly was previously reported in an earlier publication on this cohort [6]. As a comparison, in a study of a cohort of 2 558 psychiatric patients about one in three patients were exposed to drugs with “known” risk for TdP and one in five patients had a co-prescription of more than one risk drug [15]. Furthermore, the study showed that an increased number of concomitantly used drugs classified as “conditional” or “known” risk classes, but not “possible” risk classes, appeared additive with regard to the QTc interval [15]. In addition, animal studies demonstrate that use of multiple drugs with QT-prolonging properties increases the risk for QT prolongation and TdP to a larger extent than use of only one QT-prolonging drug [11]. An increased risk for TdP could also be due to concomitant use of drugs that affect the same metabolic pathway which can result in elevated plasma levels of a drug.

Prior to the occurrence of TdP, almost half of the patients in our study cohort had diagnoses associated with increased risk for arrhythmia. A substantial proportion of patients with risk diagnoses also used risk drugs. Certain conditions and drugs can prolong the action potential, enhance early afterdepolarizations and thereby increase the risk for TdP. Impaired renal or hepatic function can result in elevated drug levels and increased risk of QT prolongation. Patients with heart failure or structural heart disease can have a diminished repolarization reserve, making them more susceptible to the arrhythmogenic effects of QT-prolonging drugs thereby facilitating the development of TdP even at therapeutic drug levels [1, 6].

Even among patients with congenital LQTS, a condition with increased risk for TdP, a significant proportion of patients (60%) were treated with risk drugs [16]. The prevalence of congenital LQTS in the literature is estimated to 1:2000 with a slight female predominance, based on data from a large cohort [17]. In comparison, in our study cohort the prevalence of a registered diagnosis of LQTS was 2% (corresponding to 40:2000).

Our study cohort of patients with first-time TdP arrhythmia had an equal proportion of females and males. We observed sex-differences in dispensed drugs and the prevalence of risk diagnoses, indicating that risk factors for TdP differed between females and males. A higher proportion of females compared to males had dispensed antidepressant drugs. Males had a higher prevalence of coexisting heart disease and other diagnoses associated with an increased risk for TdP. However, females had a higher prevalence of LQTS even though the total number of patients with LQTS was too small to allow any firm conclusions.

### Individualized and safer prescribing of drugs based on CDSS tools

Although several drugs associated with risk for TdP have been withdrawn from the market during the last decades recent examples highlight the need for caution concerning new drugs. One example is the increasing number of anticancer drugs with new signals of potential drug-induced long QT syndrome observed in the international pharmacovigilance database VigiBase [18]. In addition, a high prevalence of use of QT-prolonging drugs and drug-drug interactions among cancer patients has been reported [19]. Another example is the use of old drugs for new indications, such as hydroxychloroquine and chloroquine during the COVID-19 pandemic. These examples emphasize the ongoing need for improved awareness among healthcare professionals, and a need for clinical decision-support tools [6].

Janusmed Risk Profile, a CDSS embedded in the electronic health record, generates alerts and assists prescribers in assessing the risk for QT prolongation in patients using several drugs. A study evaluating drug prescription patterns before and after its introduction showed a pronounced reduction in co-prescription of QT prolonging drugs [20]. Another study highlighted that alerts were more likely to be followed when the CDSS also provided safer therapeutic alternatives [21]. However, using a CDSS can also lead to over alerts. In one study nearly half of physicians reported that the TdP warning system and the advisory appeared too often [22]. A risk prediction model that incorporated both patient-specific risk factors, exposure to QT-prolonging drugs and severity of interactions between QT-prolonging drugs, outperformed a CDSS based solely on drug-drug interactions alerts. Specifically, use of antiarrhythmics, age and baseline QTc interval were the most important predictors for the risk of QT prolongation for a particular drug pair [23].

In our study, most patients with a registered TdP diagnosis had been dispensed drugs with risk for QT prolongation and/or had comorbidities associated with risk for TdP. These findings reinforce the need to integrate both individual patient characteristics and information on risk drugs when assessing the risk for TdP in a specific patient.

### Strengths and limitations

The registers used in our study cover the whole Swedish population and include a large number of patients with a registered diagnosis of TdP. The study included diagnoses in hospital care and all dispensed drugs. Additional strengths include an analysis of the classification of medications using two distinct CDSS and an analysis of comorbidities. A combination of drugs resulting in pharmacokinetic drug interactions and increased plasma levels of risk drugs was not analysed in this study but is another factor important to acknowledge in the clinical setting.

Using data on dispensed drugs was a proxy for medicine use and should be a more accurate representation of medication use compared to prescription data. Dispensed prescribed medications might have overestimated the exposure to medications, i.e. that patients were exposed when truly unexposed [8]. Data on drugs dispensed within 3 months prior to TdP diagnosis might have both underestimated and overestimated true drug use. Some drugs may still have been used though dispensed more than 3 months earlier. Other drugs might have been used for only a short period of time prior to TdP (i.e. antibiotics). Moreover, drugs used in hospitals and drugs purchased over the counter were not included as they are not registered in the SPDR.

The cohort includes only a subset of individuals affected by TdP arrhythmia since an ECG is required for diagnosis. Thus, the study cohort is only representative for patients with diagnosed TdP, most of them being in hospital care. We did not have access to diagnoses from primary care since the NPR only covers hospital and specialized outpatient care and only diagnoses 5 years prior to TdP were included. This could lead to an underestimation of the number of patients with risk diagnoses.

Furthermore, we did not include electrolyte disturbances among the diagnoses. However, we believe that electrolyte abnormalities are underreported as ICD diagnosis unless they are pronounced. The date of hospital admission was used as the date for TdP even if the arrhythmia had occurred during the hospital stay. This could have influenced the analysis of number of days between TdP occurrence and death.

### Conclusions and implications

A majority of patients in our study had both risk drugs and risk diagnoses for arrhythmia prior to the occurrence of TdP arrhythmia. This highlights the importance of access to Clinical Decision Support Systems (CDSSs) to assist prescribers in evaluating the risk of arrhythmia regarding individual drugs and their combinations. Patient specific factors, such as underlying risk diagnoses and sex, should also be incorporated into the risk assessment for TdP.

## Supplementary information

ESM 1 (DOCX 21 kb)

## Data Availability

All data supporting the findings of this study are available within the paper and its Supplementary Information.

## References

[1] Tisdale JE, Chung MK, Campbell KB, Hammadah M, Joglar JA, Leclerc J et al (2020) Drug-induced arrhythmias: a scientific statement from the American Heart Association. Circulation 142(15):e214–ee33 32929996 10.1161/CIR.0000000000000905

[2] Al Sayed ZR, Pereira C, Le Borgne R, Viaris de Lesegno C, Jouve C, Penard E et al (2024) CAVIN1-mediated hERG dynamics: a novel mechanism underlying the Interindividual variability in drug-induced long QT. Circulation 150(7):563–576 38682330 10.1161/CIRCULATIONAHA.123.063917

[3] QTdrugs List [Internet]. AZCERT, Inc. 1822 Innovation Park Dr., Oro Valley, AZ 85755v. [cited 2020-12-01]

[4] Bottiger Y, Laine K, Korhonen T, Lahdesmaki J, Shemeikka T, Julander M et al (2018) Development and pilot testing of PHARAO-a decision support system for pharmacological risk assessment in the elderly. Eur J Clin Pharmacol 74(3):365–371 29198061 10.1007/s00228-017-2391-3 PMC5808089

[5] Danielsson B, Collin J, Jonasdottir Bergman G, Borg N, Salmi P, Fastbom J (2016) Antidepressants and antipsychotics classified with torsades de pointes arrhythmia risk and mortality in older adults - a Swedish nationwide study. Br J Clin Pharmacol 81(4):773–783 26574175 10.1111/bcp.12829 PMC4799929

[6] Danielsson B, Collin J, Nyman A, Bergendal A, Borg N, State M et al (2020) Drug use and torsades de pointes cardiac arrhythmias in Sweden: a nationwide register-based cohort study. BMJ Open 10(3):e034560 32169926 10.1136/bmjopen-2019-034560 PMC7069257

[7] Ludvigsson JF, Andersson E, Ekbom A, Feychting M, Kim JL, Reuterwall C et al (2011) External review and validation of the Swedish national inpatient register. BMC Public Health 11:450 21658213 10.1186/1471-2458-11-450 PMC3142234

[8] Wettermark B, Hammar N, Fored CM, Leimanis A, Otterblad Olausson P, Bergman U et al (2007) The new Swedish prescribed drug register--opportunities for pharmacoepidemiological research and experience from the first six months. Pharmacoepidemiol Drug Saf 16(7):726–735 16897791 10.1002/pds.1294

[9] Brooke HL, Talback M, Hornblad J, Johansson LA, Ludvigsson JF, Druid H et al (2017) The Swedish cause of death register. Eur J Epidemiol 32(9):765–773 28983736 10.1007/s10654-017-0316-1 PMC5662659

[10] Hammar T, Jonsen E, Bjorneld O, Askfors Y, Andersson ML, Lincke A (2024) Potential adverse drug events identified with decision support algorithms from Janusmed risk profile-a retrospective population-based study in a Swedish region. Pharmacy 12(6) 10.3390/pharmacy12060168 PMC11587405 39585094

[11] Frommeyer G, Fischer C, Ellermann C, Dechering DG, Kochhauser S, Lange PS et al (2018) Additive Proarrhythmic effect of combined treatment with QT-prolonging agents. Cardiovasc Toxicol 18(1):84–90 28612303 10.1007/s12012-017-9416-0

[12] Zeltser D, Halkin A, Prokhorov V, Heller K, Viskin S, de Pointes T (2003) Due to noncardiac drugs. Most patients have easily identifiable risk factors. Medicine 82(4):282–290 12861106 10.1097/01.md.0000085057.63483.9b

[13] Sarganas G, Garbe E, Klimpel A, Hering RC, Bronder E, Haverkamp W (2014) Epidemiology of symptomatic drug-induced long QT syndrome and torsade de pointes in Germany. Europace 16(1):101–108 23833046 10.1093/europace/eut214

[14] Then MI, Tumena T, Sledziewska A, Gassmann KG, Maas R, Fromm MF (2023) Development in prescriptions of contraindicated and potentially harmful QT interval-prolonging drugs in a large geriatric inpatient cohort from 2011 to 2021. Clin Pharmacol Ther 113(2):435–445 36471654 10.1002/cpt.2813

[15] Meid AD, Bighelli I, Machler S, Mikus G, Carra G, Castellazzi M et al (2017) Combinations of QTc-prolonging drugs: towards disentangling pharmacokinetic and pharmacodynamic effects in their potentially additive nature. Ther Adv Psychopharmacol 7(12):251–264 29201344 10.1177/2045125317721662 PMC5676495

[16] Weeke PE, Kellemann JS, Jespersen CB, Theilade J, Kanters JK, Hansen MS et al (2019) Long-term proarrhythmic pharmacotherapy among patients with congenital long QT syndrome and risk of arrhythmia and mortality. Eur Heart J 40(37):3110–3117 31079148 10.1093/eurheartj/ehz228

[17] Krahn AD, Laksman Z, Sy RW, Postema PG, Ackerman MJ, Wilde AAM et al (2022) Congenital long QT syndrome. JACC Clin Electrophysiol 8(5):687–706 35589186 10.1016/j.jacep.2022.02.017

[18] Salem JE, Nguyen LS, Moslehi JJ, Ederhy S, Lebrun-Vignes B, Roden DM et al (2021) Anticancer drug-induced life-threatening ventricular arrhythmias: a World Health Organization pharmacovigilance study. Eur Heart J 42(38):3915–3928 34370839 10.1093/eurheartj/ehab362 PMC8677441

[19] Khan Q, Ismail M, Khan S (2017) Frequency, characteristics and risk factors of QT interval prolonging drugs and drug-drug interactions in cancer patients: a multicenter study. BMC Pharmacol Toxicol 18(1):75 29191244 10.1186/s40360-017-0181-2 PMC5710059

[20] Petersson L, Schorgenhofer C, Askfors Y, Justad H, Dahl ML, Andersson ML (2023) Pharmacological risk assessment among older patients with polypharmacy using the clinical decision support system Janusmed risk profile: a cross-sectional register study. Drugs Aging 40(4):369–376 37039960 10.1007/s40266-023-01021-9 PMC10113338

[21] Trinkley KE, Pell JM, Martinez DD, Maude NR, Hale G, Rosenberg MA (2021) Assessing prescriber behavior with a clinical decision support tool to prevent drug-induced long QT syndrome. Appl Clin Inform 12(1):190–197 33694143 10.1055/s-0041-1724043 PMC7946597

[22] Gallo T, Heise CW, Woosley RL, Tisdale JE, Antonescu CC, Gephart SM et al (2022) Clinician satisfaction with advanced clinical decision support to reduce the risk of Torsades de Pointes. J Patient Saf 18(6):e1010–e10e3 35238815 10.1097/PTS.0000000000000996

[23] Muylle KM, van Laere S, Pannone L, Coenen S, de Asmundis C, Dupont AG et al (2023) Added value of patient- and drug-related factors to stratify drug-drug interaction alerts for risk of QT prolongation: development and validation of a risk prediction model. Br J Clin Pharmacol 89(4):1374–1385 36321834 10.1111/bcp.15580

