# Supplementary material for: Prescribed drugs and comorbidities as risk factors for Torsades de Pointes arrhythmia: a Swedish population-based cohort study
Source: Eur J Clin Pharmacol. 2026 Jan 19;82(2):57. doi: 10.1007/s00228-025-03990-9 (PMC12816018; doi:10.1007/s00228-025-03990-9)
Supplement: Supplementary file 1 — (DOCX 21 kb) [file 228_2025_3990_MOESM1_ESM.docx]

**Supplementary material**

**Prescribed Drugs and Comorbidities as Risk Factors for Torsades de Pointes Arrhythmia: A Swedish Population-Based Cohort Study**

Marine L. Andersson^1^, Johan Fastbom^2^, Bengt Danielsson^1^, Eva Wikström ^1^, Marja-Liisa Dahl^1^, Karolina Nowinski^1^

**Running heading:** Risk Factors for Torsades de Pointes arrhythmia

**^1^ Department of Laboratory Medicine and Department of Clinical Pharmacology, Karolinska Institutet and Karolinska University Hospital, Stockholm, Sweden**

**^2^ Aging Research Center, Karolinska Institutet and Stockholm University, Stockholm, Sweden**

**Corresponding author:**Karolina Nowinski, M.D, Ph.D
Department of Clinical Pharmacology C1:68
Karolinska University Hospital
141 86 Stockholm, Sweden
karolina.nowinski@regionstockholm.se

**Supplementary Table 1 – Diagnoses (ICD-10) used to define the population.**

| **Diagnosis** | **ICD 10** |
| --- | --- |
| Acute coronary syndrome | I21, I22, I23, I24 |
| Angina pectoris | I20 |
| Atrial fibrillation or flutter | I48, I49 |
| AV block II | I441 |
| AV block II and III | I441, I442, I443 |
| AV block III | I442 |
| Cardiac arrest | I46 |
| Cardiomyopathies | I42, I43 |
| Cerebrovascular disease | I60-I69 |
| Chronic coronary syndrome | I25 |
| Chronic kidney disease | N17-N19 |
| Chronic obstructive pulmonary disease | J44 |
| Fascicular block, bundle-branch block, intraventricular block, pre-excitation | I44.4-I44.7, I45 |
| Heart failure | I50 |
| Heart valve disease | I340-I341, I350-I352, I359, I361, I369 |
| Hypertension | I10-I15 |
| Liver disease | K70-K77 |
| Long QT Syndrome (LQTS) | I498E |
| Paroxysmal tachycardia | I47.9 |
| Poisoning | T36-T65 |
| Premature depolarization | I49.1-I49.4 |
| Reentry ventricular arrhythmia and/ or paroxysmal ventricular tachycardia | I47.0, I47.2 |
| Renal failure | N00-N08, N10-N16 |
| Rheumatic diseases | M05-M14, M30-M36 |
| Sepsis | A021, A402, A403, A408-A412, A415, A418, A419, B377, R572 |
| Sick sinus syndrome | I49.5 |
| Supraventricular arrhythmia | I47.1 |
| Unspecified arrhythmia | I479, I498, I498A, I499, |
| Vascular disease | I70-I79 |
| Ventricular arrhythmia | I470, I472A, I472B, I49.0 |
| Ventricular fibrillation | I49.0 |
| Ventricular premature depolarization | I493 |

**Supplementary Table 2. Diagnoses (ICD-10) registered at the time of hospitalization for TdP arrhythmia**Diagnoses at the time of hospital care episode of TdP (n = 762). The numbers refer to number of individuals. N/a denotes that diagnosis occurred in less than 5 individuals.

| **Comorbidities at the time of TdP** | **Women n=383** | **Men n=379** | **All n=762** |
| --- | --- | --- | --- |
| Atrial fibrillation or flutter | 15 | 23 | 38 |
| Hypertension | 25 | 32 | 57 |
| Heart failure | 22 | 21 | 43 |
| Cardiomyopathies | n/a | n/a | 13 |
| Chronic coronary syndrome | 8 | 21 | 29 |
| Acute coronary syndrome | 11 | 41 | 52 |
| Angina pectoris | n/a | n/a | 7 |
| Heart valve disease | n/a | n/a | 11 |
| Cerebrovascular diseases | 7 | 5 | 12 |
| Renal failure | n/a | n/a | 16 |
| Cardiac arrest | 6 | 14 | 20 |
| Ventricular fibrillation | 5 | 11 | 16 |
| Supraventricular or unspecified arrhythmia | n/a | n/a | 8 |
| Ventricular premature depolarization | n/a | n/a | 9 |
| AV block III | 7 | 11 | 19 |
| AV block II | n/a | n/a | 5 |
| Sick sinus syndrome | n/a | n/a | 6 |

**Table 3. The 25 most dispensed drugs before the occurrence of Torsade de Pointes arrhythmia (TdP) among the 762 patients.**

| **Substance** | **All N (%)** | **Females n(%)** | **Males n(%)** |
| --- | --- | --- | --- |
| acetylsalicylic acid | 218 (29) | 109 (28) | 109 (29) |
| furosemide | 203 (27) | 109 (28) | 94 (25) |
| metoprolol | 183 (24) | 74 (19) | 109 (29) |
| paracetamol | 181 (24) | 118 (31) | 63 (17) |
| simvastatin | 141 (18) | 58 (15) | 83 (22) |
| enalapril | 112 (15) | 50 (13) | 62 (16) |
| omeprazole | 110 (15) | 54 (14) | 56 (15) |
| warfarin | 106 (14) | 45 (12) | 61 (16) |
| zopiclone | 96 (13) | 57 (15) | 39 (1) |
| cyanocobalamine | 81 (11) | 45 (12) | 36 (9) |
| citalopram | 80 (10) | 58 (15) | 22 (6) |
| bisoprolol | 66 (9) | 37 (10) | 29 (8) |
| digoxin | 68 (8) | 30 (8) | 38 (10) |
| levothyroxine | 61 (8) | 48 (13) | 13 (3) |
| atorvastatin | 59 (8) | 21 (5) | 38 (10) |
| glyceryltrinitrate | 57 (8) | 27 (7) | 30 (8) |
| isosorbide mononitrate | 58 (8) | 29 (8) | 29 (8) |
| oxazepam | 56 (7) | 37 (10) | 19 (5) |
| amlodipine | 55 (7) | 27 (7) | 26 (7) |
| candesartan | 53 (7) | 25 (7) | 28 (7) |
| spironolactone | 52 (7) | 22 (6) | 30 (8) |
| felodipine | 51 (7) | 23 (6) | 28 (7) |
| metformin | 49 (7) | 18 (5) | 31 (8) |
| losartan | 49 (6) | 25 (7) | 24 (6) |
| allopurinol | 47 (6) | 17 (4) | 30 (8) |
